# “Anything Can Happen Here”: Mother–Child Experiences Navigating Life as Residents of an Urban Red‐Light Brothel District in India

**DOI:** 10.1111/famp.70034

**Published:** 2025-03-30

**Authors:** Rochelle L. Dalla, Sharvari Karandikar, Ravi Chavan

**Affiliations:** ^1^ Child, Youth & Family Studies University of Nebraska–Lincoln Lincoln Nebraska USA; ^2^ College of Social Work The Ohio State University Columbus Ohio USA; ^3^ Social Worker and Activist Mumbai India

**Keywords:** brothel, brothel‐based children, female sex workers, red‐light district

## Abstract

Female sex workers as mothers are disproportionately disadvantaged and experience myriad intersecting vulnerabilities, including poverty, physical and mental health challenges, limited access to health care and health care providers, stigma and discrimination, substance use, and histories of trauma. These risks elevate the potential for negative developmental outcomes for their children. To date, little information exists on the contextual and familial dynamics of female sex workers and their children. Yet, this information is critical for providing effective, evidence‐informed interventions. This is a life‐course developmental examination of female sex workers and their children residing in an urban brothel district in India, framed in ecological systems theory. From mothers, we were particularly interested in developmental trajectories that led to commercial sex work, intergenerational family dynamics, microsystems of mothers and their children (residence patterns), and concerns for child wellbeing given environmental dangers of the red‐light areas. Inquiries with children were aimed at understanding the family microsystem—dynamics and residence, other influential microsystems (peer and school), as well as the larger red‐light district neighborhood (exosystem) and future aspirations. Most mothers had been trafficked into the sex industry. Because of their work, few remained in contact with families of origin. Mothers' concerns included generating income, getting children educated, and returning children to hostels. Prior to the pandemic, most child participants lived in hostels rather than the red‐light area and described it as dirty and unsafe. Children described types of social support given and received by mothers and prioritized education. Continued policy and research that explore innovative measures for limiting social disparities in educational attainment for vulnerable children (e.g., mobile school programs) are recommended.

## 1 Introduction

Estimating the global size of the population of female sex workers (FSWs) is extremely difficult, if not impossible (Sabin et al. 2016). Estimating the number of FSWs engaged in a particular *type* of sex work (street and brothel based^1^) and who are also mothers, with any degree of certainty, is even more daunting. The sex market operates along a continuum of organization, complexity, and varying levels of vulnerability and exploitation (Kjellgren 2024). Street‐ and brothel‐based FSWs are unique because they work in environments that place them at the greatest risk for physical harm, while simultaneously earning the least amount of money for their sexual labor compared to many—if not most—other types of FSWs (e.g., call girls, internet‐based sex workers, and other in‐door and protected environments; Beattie et al. 2016; Goldenberg et al. 2018; West et al. 2021). That said, perhaps knowing the number of mothers engaged in the street‐ and brothel‐based sex trade is much less important than understanding the unique vulnerabilities to which they, and their children, are exposed. Indeed, given precarious family, environmental, social, and cultural environments, street‐ and brothel‐based FSWs are disproportionately disadvantaged (Dewey et al. 2018; Duff et al. 2014)—a fact which elevates risk for their children as well (Beard et al. 2010; John‐Fisk 2013; Willis et al. 2016; Willis et al. 2014).

Although a great amount of information has been garnered on the particular vulnerabilities of street‐ and brothel‐based FSWs, most of this research focuses on either HIV risk or their dual identities as mothers and persons engaged in the commercial sex industry. Substantially less attention has been paid to FSWs' relationships with their children. Further, significant contradictions exist in the data that are available. Yet, the mother–child relationship is crucial for a child's social–emotional development (Bianciardi et al. 2023)—and can serve as a protective factor and source of resilience that enhances positive child outcomes (Yoon 2022). On the other hand, what is also clear in the extant literature is that trauma can have a profound impact on parenting practices and, by extension, the parent–child relationship (e.g., Iyengar et al. 2014). Thus, there is a need to better understand the family dynamics and life experiences among a particularly vulnerable and marginalized population.

Grounded in ecological systems theory (Bronfenbrenner 1977), this investigation presents data gathered from both brothel‐based FSWs and their children. To this end, interviews with mothers were framed to reveal: (1) developmental circumstances that led to entry into the commercial sex industry; (2) family and work microsystems, and linkages across those; and (3) aspirations for children's futures. Inquiries with children were aimed at understanding (1) microsystem (family, school) dynamics and social relations, (2) linkages across those, and (3) future aspirations. The complementary data provide insight into the unique contextual and relational dynamics between brothel‐based FSWs and their children.

### 1.1 Female Sex Workers as Mothers

Since the early 2000s, investigations of street‐ and brothel‐based FSWs *as mothers* have expanded appreciably. Taken together, the literature reveals a singular common thread: FSWs as mothers experience myriad *intersecting vulnerabilities* (Karandikar and Prospero 2010; Karandikar et al. 2022), including poverty, compromised physical and mental health, limited access to health care and health care providers, stigma and discrimination, substance use, and histories of trauma (Bletzer 2005; Dalla 2003; Dalla and Kreimer 2017; Dewey et al. 2018; Joffres et al. 2008; McClelland and Newell 2008; Peled and Parker 2013; Reed et al. 2013; Willis et al. 2014; Zalwango et al. 2010). Many are also migrants, which further elevates vulnerability because of potential language and culture barriers, as well as precarious legal status (Dasgupta 2023; Ramanaik et al. 2012; Walker et al. 2017). Noteworthy too is that, in India, FSWs are almost always—especially if they are Hindu, members of a *Dalit* caste,^2^ whose entry into the sex trade was either forced or coerced and who lacked other viable options for generating income (Jha and Sharma 2016; Saggurti et al. 2015). In the growing body of research addressing FSWs as mothers, two prominent lines of inquiry exist, including: (1) risk and vulnerabilities for HIV or other sexually transmitted infections (Beckham et al. 2015; Basu and Dutta 2011; Decker et al. 2015; Duff et al. 2014; Papworth et al. 2015; Reed et al. 2013; Rolon et al. 2013; Wong et al. 2012), and (2) managing dual (and often competing) identities as mothers and participants in the commercial sex trade (e.g., Basnyat 2020; Bjønnes 2015; Couvrette et al. 2016; Dodsworth 2014; Ma et al. 2019; Nyariki et al. 2023). Far fewer investigations have centered on the parent–child relationship or parenting strategies used to safeguard children.

#### 1.1.1 Child‐Rearing Practices

In an early investigation, Pardeshi and Bhattacharya (2006) examined child‐rearing practices among brothel‐based FSWs in Pune, India, by interviewing Devadasi^3^ (*n* = 25) migrants from rural villages, migrants from Nepal (*n* = 20), and migrants from other Indian states (*n* = 15). Child‐rearing practices were constructed in response to the availability and quality of familial support. Devadasis, who maintained strong relationships with families of origin in their native villages, were able to leave the sex industry early in their pregnancies, return to natal villages, and then return to work with children left in the care of kin. Alienated from families of origin, the other participants were forced to work late into their pregnancies and to resume work shortly after giving birth. As their children aged, mothers often relied on nonkin placements (e.g., hostels, rehabilitation centers) to shield them from the red‐light areas. This research parallels results from other studies in many aspects, including the impact of familial support on child‐rearing practices and maternal strategies used to protect children from harm (i.e., sending them to live in hostels, sending children to natal villages to be cared for by kin) even if those strategies limited mother–child physical contact (Dalla et al. 2019; McCloskey et al. 2021).

Other studies document FSWs as caring mothers who sacrifice for their children, prioritize children's needs, and make decisions about their work based on children's well‐being (Beckham et al. 2015; Praimkumara and Goh 2016). Basu and Dutta (2011), for instance, summarized results of interviews with 23 mothering FSWs in the red‐light area of Kalighat (Kolkata, India), writing,

The mothers hoped that their children would be educated, because education, they communicated, would help the children negotiate a life of respectability outside sex work. It was their dream and hope for their children that catalyzed the enactment of responsibilities and duties that came with motherhood. (p.115)

Conversely, research produced by others (Bjønnes 2015; Bogart et al. 2005; Dalla 2003, 2004; McClelland and Newell 2008) characterizes many FSWs as having little desire or ability to care for their children, and further, as parents who often voluntarily relinquish, or lose, custody of children due to ongoing abuse, neglect, failure to protect, or other precarious living conditions (e.g., domestic violence). The primary difference in these deeply conflicted narratives (i.e., FSWs as mothers who strive to protect their children from harm and engage in meaningful child‐rearing practices versus those who do not) appears to rest on whether or not the FSWs in question abuse substances (e.g., crack cocaine, heroin, methamphetamines), on the one hand, and whether or not sex work is a means of earning money to support addictions, on the other. This distinction should not be minimized. Sex work in many parts of the world—and especially LMICs (lower‐ and middle‐income countries) of the global south—is often fueled by subsistence living and a means for survival, whereas sex work (specifically street‐based sex work) in Western and more affluent countries of the global north is typically associated with substance abuse. Addiction impacts relationships—and can have dire consequences for the mother–children relationship—whether or not the mother is also involved in the commercial sex trade (Martin 2011; Parolin and Simonelli 2016).

### 1.2 Vulnerabilities of Children of Female Sex Workers

Children of FSWs have only recently emerged as a focal point in research. Still, regardless of the geographic origination of the study, research questions posed, methodological approach, or age of the participants, children of FSWs emerge as a uniquely disadvantaged population at risk for myriad physical, social, and emotional challenges. In her writing of brothel‐based children specifically, Rath (2020) paints a particularly grim perspective:

…the [brothel] itself as consisting of 10 or more women including children huddled into a small dark room where the women entertain their customers keeping their children under the bed. The room usually stinks of semen…[with an equally filthy bathroom] that stinks of human excreta […] where children mostly grow loitering around the streets, sleeping on the pavement, joining gambling groups, helping agencies and traffickers procure drugs, or running errands for their mothers' customers [resulting in] drastic psychological and emotional pressures leading many to become rebellious, abusive and violent, and to develop feelings of bitterness towards life. (p. 332)

Rath's account is especially provocative and meant to ire the Indian Government, and especially the Ministry of Women and Child Development, into action. In an investigation of threats to the health and welfare of children of brothel workers in Bangladesh, Willis et al. (2014) summarize how stigma and discrimination compromise access to safe housing, health care, education, and law enforcement protection and that these children are at risk for sexual and labor exploitation as well as trauma from witnessing brutality against their mothers. These experiences, conclude the authors, negatively alter these children's life trajectories. Heightened risk exposure is not unique to children of FSWs in South Asian countries. In a scoping review, Beard et al. (2010) compared vulnerabilities faced by children of FSWs with children of drug users. All experienced stigma and discrimination; vulnerabilities unique to children of FSWs included separation from parents, sexual abuse, early sexual debut, introduction to sex work in adolescence, low school enrollment, psychosocial trauma, and social marginalization. Similar results are echoed by others. Ugandan Children of FSWs reported statistically significant rates of each type of victimization examined (e.g., emotional abuse, sexual victimization, witness to domestic violence), in comparison to a comparable sample of at‐risk youth without a parent involved in the commercial sex trade (Ouma et al. 2023). Finally, on the US–Mexico border, Servin et al. (2015) found that 20% of their sample of 628 drug‐injecting FSWs reported having a parent—88% of the time a mother—also involved in the sex trade. Threats to well‐being among this subset (*n* = 125) also included, for instance, injecting drugs prior to age 18 (31%), working in the sex trade prior to age 18 (33%), rape as a minor, and injecting drugs within the past month.

### 1.3 Children's Voices

To date, only a handful of qualitative studies have included minor children of FSWs as research participants. Nadarajah and Fadzil (2015) interviewed 10 children of FSWs in Kuala Lumpur, Malaysia, to identify their “experiences, challenges and needs” (p. 57). Educational difficulties were particularly acute. Most were illiterate due to large class sizes and lack of teachers in schools, difficulty concentrating due to their family and housing conditions, and language barriers. Although they tried to hide their mothers' occupations from others out of fear of discrimination, the children were also protective of their mothers and concerned for their physical and emotional well‐being.

Education is a human right of childhood and critical for upward mobility. It is therefore not surprising that most qualitative studies with children of FSWs focus on this aspect of their lives. Shohel (2013) conducted interviews and focus groups with 30 children of FSWs in Bangladesh to identify challenges encountered in school. Although the participants performed well academically and enjoyed going to school viewing it as a place where they could “learn many things and play with friends” (p. 8), they also faced adversities including prejudice from teachers and peers, and difficulty at home finding someone to assist with schoolwork (i.e., due to mothers' low levels of education). The results of this study mirror similar investigations in Mumbai (John‐Fisk 2013) and North Bengal (Topno and Sarkar 2021), India. Finally, peer bullying—often due to mother's occupation—has been identified in several studies (Dutt et al. 2017; Jannat Nijhu and Letchamanan 2022) and is considered a primary motive for helping the children of FSWs enrolled in residential schools—a place free of negative peer taunting as well as the dangers of red‐light areas where mothers often reside.

### 1.4 Resilience and Resources

According to the American Psychological Association (APA), resilience is the process and outcome of successfully adapting to difficult or challenging life experiences (APA, forthcoming). Given the adverse circumstances often imposed upon many FSWs and their children, resilience is essential for optimal developmental outcomes and can be cultivated through resources and skills. Social networks are often cited as important resources for informational, tangible, and emotional support for both FSWs and their children (Basu and Dutta 2011; Dutt et al. 2021; Goh and Praimkumara, 2015). In fact, a recent investigation highlights the necessity of social capital in helping shelter‐based children of FSWs overcome social exclusion (Rahman et al. 2023).

National and international nongovernmental organizations (NGOs and INGOs) exist across the globe—from Bangladesh to Zimbabwe—aimed at providing support and resources to FSWs and their children (Beard et al. 2010; Dalla et al. 2019; Yerpude & Jogdand; 2012). These sources typically provide information, education assistance, skill development, and access to health care and safe shelter—and are particularly crucial for survival during times of crises (e.g., the global pandemic). Resilience has been discussed in relation to mothers who are FSWs. Especially important here is research documenting formal support programs that allow FSWs to find employment, complete drug treatment, and exit the sex industry (Baker et al. 2010; Dalla 2006; Goh and Praimkumara, 2015; Rolon et al. 2013).

Noteworthy, and in response to reports of bleak circumstances and dire needs of children of FSWs which currently overwhelm the literature, Sircar and Dutta (2011) provide a contrasting perspective. They introduce Amra Padatik (or AP; “We are Foot Soldiers”), a collective formed in 2005 by children of sex workers in the red‐light district of Sonagachi (Kolkata, India). AP's mission is to gain dignity for FSW mothers and claim personal rights as children of sex workers. Sircar and Dutta (2011) interviewed the founding members who described an objective of replacing the traditional 3Rs (raid, rescue, rehabilitation) with transformative counter Rs (resilience, reworking, and resistance). Sircar and Dutta (2011), 334) write,

While these children are at the receiving end of disadvantage – because of the locations where they live, the profession of their mothers, as well as being burdened by the hierarchy of age – they are also active agents in resisting disadvantage and formulating daily negotiation strategies which are an exemplary show of resilience.

Although not intended to be characteristic of the experiences of all children in Sonagachi, Sircar and Dutta (2011) nonetheless highlight the need to be mindful of heterogeneity in experiences of, and reactions to, adversity as well as of our own assumptions and biases as researchers.

### 1.5 Theory

Revolutionary when it emerged, and still instructive and relevant 60+ years later, bio‐ecological systems theory (or EST; Bronfenbrenner 1977, 2005) provided the theoretical guidance for this investigation. Visually, EST situates the developing person in the center of a nested series of concentric circles—each representing unique social, physical, and cultural contexts that impact development through time. In this study, we focus on the unique and overlapping developmental trajectories of two individuals: namely, mothers and their children. EST emphasizes interaction between each person (i.e., including all physical and constitutional characteristics) and their unique sociocultural environment (including historical events and processes) for molding developmental outcomes. Thus, the developmental trajectories of mothers (e.g., assessment of historical patterns that led to sex work and familial dynamics in family of origin), as well as future aspirations (e.g., for children), are important.

Although the theory contains five systems, four (i.e., the microsystem, mesosystem, exosystem, and macrosystem) are particularly relevant to this investigation. The microsystem refers to the physical spaces and complex social interactions in immediate settings containing the individual (Bronfenbrenner 1977). In this study, we focus specifically on immediate family and residence patterns of mothers and children, mothers' work, and children's school. Mesosystems (interrelations or linkages across microsystems; Bronfenbrenner 1977) were examined between family–school (for both mother and child) and family–hostel (child), as well as between immediate family and extended kin in rural villages (for mother and child). The exosystem refers to linkages between two or more settings, one of which may not contain the developing person but affects them nonetheless. That is, a developing person can be strongly influenced by people and places that they may not directly interact with, including their parents' workplace, neighborhood, and extended family. In this investigation, we focused specifically on brothel areas in which FSW mothers work and the red‐light areas in which the mothers and children were living as having enormous impact on children's development. Finally, the macrosystem refers to the blueprint of society and encompasses political, economic, and cultural patterns that impact individuals' micro‐, meso‐, and exosystems. In direct relation to the macrosystem is gender, class, and caste discrimination—factors frequently associated with unmarried, Dalit (lowest caste) women's entry into the commercial sex trade (Dalla and Kreimer 2017; Dandona et al. 2006; Dasgupta 2023; Swathisha and Sibnath 2022) which also accounted for questions relating to historical processes leading to the adult participants' entry into the sex trade; and the India government's decision to shutter residential schools leading to children's residence with mothers in red‐light brothel areas, setting in motion a host of micro‐, meso‐, and exosystemic issues investigated herein.

### 1.6 Study Purpose

This investigation helps fill gaps in current understanding of family dynamics of a uniquely marginalized population. Capitalizing on EST, this investigation was designed to examine developmental trajectories of FSW mothers and their children, with particular focus on unique and overlapping microsystems. To this end, interviews with mothers were framed to reveal: (a) developmental circumstances that led to entry into the commercial sex industry; (b) family and work microsystems, and linkages across those; and (c) aspirations for children's futures. Inquiries with children were aimed at understanding (a) microsystem (family, school) dynamics and social relations, (b) linkages across those, and (c) future aspirations.

## 2 Method

This investigation was aimed at understanding the lived experiences and developmental trajectories of FSWs and their children residing in a brothel‐based urban red‐light area of India. In‐depth personal interviews allow the to delve deeply into social and personal matters (e.g., developmental histories and familial dynamics), while simultaneously affording privacy (vs. group interviews) when discussing sensitive issues (Bayeck 2021) and thus, were deemed relevant for the purposes of this study. The first two authors of this study were prepared to travel to Mumbai from our United States–based institutions for data collection. However, in March 2020, the entire country went under lockdown, and travel into or out of India was impossible. Methodological adaptations were common over the course of the global pandemic (Cornejo et al. 2023; Jung et al. 2021; Palese et al. 2023) and this study was no exception. Rather than wait out an uncertain length of time, we hired and trained a Mumbai‐based research assistant (RA, third author) to assist. The RA was selected because he was (a) bilingual (Hindi/English); (b) a community‐based social worker with over two decades of experience working with FSWs; and (c) had prior and extensive experience collaborating with the second author. The second author trained the RA in conducting in‐person interviews, using the interview protocol, and recording and uploading interview data. Interviews were completed in March 2021; all were audio‐recorded with participant permission, and all research activities were approved by the Institutional Review Boards of the University of Nebraska‐Lincoln and the Ohio State University (lead authors' affiliations), including approval to collect data from minors.

### 2.1 Methodological Rigor

Multiple strategies ensured methodological rigor, including journal writing, maintaining a detailed audit trail, reflexivity, and peer debriefing. The RA maintained a detailed audit trail during the data collection process; critical decisions regarding data collection activities were noted and reported in detail. Additionally, the RA maintained a journal in which reflections and critical learnings were documented. The second author engaged in daily peer debriefing, via zoom, with the RA to discuss the data collection process, reflections, challenges, or any other issues relevant to data collection.

### 2.2 Context

Hanuman Tekdi (HT) is a red‐light brothel area located in Bhiwandi (north of Mumbai). Similar to the infamous Mumbai red‐light areas known as Kamathipura and Falkland Road, Hanuman Tekdi is characterized by densely populated lanes, dilapidated structures, poor‐quality drinking water, and largely unsanitary living conditions. In HT, there are no public health centers. Instead, residents must rely on private clinics at their personal expense (News 18, 2020). Most FSWs in HT are migrants from rural villages in Maharashtra or other states in the country, as well as from Nepal and Bangladesh (GaonConnection 2020). FSWs in HT reside in rented rooms at a monthly cost of about 2000₹–4000₹ ($24–$48), which comprise a significant portion of their typical monthly earnings (i.e., about 10,000₹–50,000₹ or $120.00–$602.00; GaonConnection 2020).

### 2.3 Sampling and Data Collection

The RA conducted daily field visits to the red‐light area of HT to interact and build rapport with potential participants. During the process of visiting and building rapport, the RA also explained the purpose of his work in the HT area (i.e., as a research assistant) and the purpose of the study. If interested in participating, the RA explained to each their rights related to participation, that they could end the interview at any point without loss of remuneration, and that they did not have to answer any questions in which they were uncomfortable. Given varying levels of literacy and to protect anonymity, participants provided verbal consent to participate. In addition to direct recruiting, snowball sampling was also used to recruit both adult and child participants. After receiving information about the research from the RA, only those individuals who volunteered to participate in the study were recruited and interviewed. Study inclusion required that participants be 18 years of age or older, cis‐gender females with children who were soliciting in the Hanuman Tekdi red‐light area. Interviews with FSWs lasted from 1 to 1.5 h (mean = 70 min) and took place in the brothel rooms where the women worked.

Child participants were required to be less than 18 years of age and currently residing with their mothers in the HT red‐light area. The RA provided information specific to the children's interviews to the mothers of the children. The mothers provided consent for their children to participate. The RA provided information on the address of the nonprofit organization (located in the heart of the HT community) to the mothers; only those children who came to the nonprofit organization, accompanied by their mother and expressing interest in the study, were interviewed by the RA. All the interviews were one‐on‐one with the RA and the child participant while mothers waited outside the office. Interviews with children lasted 30 min to 1 h. FSWs received 1000₹ (about US$13) for participation; children received 500₹ (about US$6.50) for participation.^4^ All interviews were conducted in Hindi; audio files were transcribed in Hindi and translated literally into English by the second author. All the transcriptions and translations were cross‐checked for accuracy by the RA. All audio recordings were deleted following transcription and were labeled with pseudonyms.

#### 2.3.1 Interview Guide

Grounded in EST (Bronfenbrenner 1977) the semi‐structured interview protocols were created to provide a developmental account of participants' lives—focusing especially on relational dynamics and critical microsystems, mesosystems, and exosystems. Interviews with adult participants began by asking about events and processes that led to entry into the sex trade (i.e., Can you tell me a little bit about how you became involved in this line of work?), followed by questions about formal and informal social support (e.g., Can you talk about the primary people you receive support from—who are they? What types of help do they give?), the neighborhood exosystem (e.g., Can you talk about the people who live around here? What changes have you experienced?), relationships with children and between children and extended family (e.g., Do your children have contact with your extended family? Can you share more about that?), and impacts of COVID‐19 (e.g., How did COVID‐19 impact work? How did it impact your children and their schooling? Can you describe those changes?).

Interviews with children focused on relationships and social support in critical microsystems (e.g., can you tell me about your relationship with your mother? Who helps you if you need assistance with homework? Can you talk to me about your friends or the people you go to for support?), as well as with extended family and connections across microsystems (e.g., How is living here in HT different from living in the hostel?), the neighborhood exosystem (e.g., Do you see your neighbors? Do you shop with your mother?), impacts of COVID‐19 on microsystems (e.g., How did Covid impact school? How has it affected your mom?), and future (educational/career) aspirations (What are your future career goals? Are academics important to you—why/why not?).

Participants' responses to questions dictated the length of time spent on the question, as well as follow‐up questions. For instance, if adults reported their children and extended family had contact, follow‐up questions about how often they were in contact and the type of contact (e.g., in person, via telephone) would be posed. Similarly, if children, for instance, reported receiving support from friends, follow‐up questions would probe those peer relationships. The goal was to assess the social and physical context of participants' lives (grounded in the theoretical lens of EST), in a structured manner, while simultaneously allowing for flexibility given unique life trajectories.

## 3 Researcher Positionality

The first two authors hold advanced degrees (lead author in human development and family studies; second author in social work) and have extensive training in theoretical and methodological perspectives relevant to these disciplines. They also bring extensive experience in public health and a long history of conducting qualitative research, including in‐depth interviews with vulnerable, cross‐cultural populations. The lead author has over two decades of experience researching fragile and marginalized populations, including survivors of sex trafficking. For the past decade, her work has focused on family‐facilitated child sex trafficking in India. The second author, a native of Mumbai, India, has direct practice experience as a social worker and community advocate for sex workers in India. This practical experience has informed and guided her work, grounding her in the community and inspiring her to amplify the voices of sex workers in advocating for their rights and safety.

As authors, we acknowledge our positionality as academics based in a Western country conducting research with a highly stigmatized population in India. We are sensitive to the deep‐rooted religious, caste, and class divisions within Indian society. Drawing from our prior social work and research experience with sex workers, we are acutely aware of the challenges faced by minoritized and stigmatized individuals in this community. Throughout this research, we engaged in reflective team discussions to better understand the cultural and historical nuances that emerged during the interviews and analysis phase. We identified and documented potential biases and knowledge gaps stemming from our diverse cultural and lived experiences. These were addressed through ongoing Zoom and phone conversations, which helped ensure methodological rigor throughout the data analysis process.

## 4 Data Analysis

Data were analyzed using thematic analysis (Braun and Clarke 2006). The approach follows a six‐step process: becoming familiar with the data, generating initial codes, searching for themes, reviewing themes, defining themes, and writing up results. This process began with the two lead authors thoroughly reading the transcripts (i.e., protocols) and making notes. An initial set of codes was created. Following discussion of initial codes, axial coding was conducted independently (Saldaña 2012) and initial themes and subthemes were identified. Following this process, the authors together reviewed and defined the themes by using constant comparative analysis to examine commonalities and contrasts across the transcripts (Padgett 2008). Memos were also used to record analytic and conceptual notes, the basis for definitional statements for codes, and to assist in reflexivity on the authors' part throughout the research process (Padgett 2008). Refer to Data S1 for resultant themes and subthemes.

## 5 Participants

Mothers (*N* = 19) ranged in age from 27 to 50 years (mean = 35.2 years) and had been engaged in the commercial sex industry for an average of 13.5 years (range = 1–35 years; SD = 7.7 years). Age of entry into the commercial sex industry (CSI) varied widely, from 15 to 31 years, with an average age of 18.9 years. The length of time participants had lived in Hanuman Tekdi ranged from 2 to 21 years (mean = 13 years)—corresponding with the average length of time engaged in the sex industry. All were migrants, most (*n =* 16) from rural areas across India (refer to Table 1); three were migrants from Nepal. The majority (*n* = 11) had been married at least once, and all of those were either divorced or separated from their spouses; eight others had not formally married, although three had a current partner.

**TABLE 1:** Mother demographics.

Variables	Total sample (*N* = 19)
Age: mean (range; SD)	35.2 years (27–50 years; 5.1 years)
Marriage:	
Divorced/separated	11
Never married	8
Has current partner	3
Children (total)	37
Children per participant: mean (range; SD)	1.7 (1–4; 2.1)
Child age: mean (range; SD)	13.3 years (7–23 years; 4.6 years)
Child sex: male/female	19/18
Children live:	
Hanuman Tekdi	10
Hostel	13
Village	14
Commercial sex industry (CSI)	
Age of entry: mean (range; SD)	18.9 years (15–31 years; 1.8 years)
Years engaged in CSI: mean (range; SD)	13.5 years (1–35 years; 7.7 years)
Home village (country/state)	
India:	
Andra Pradesh	1
Bihar	1
Karnataka	8
Jharkhand	1
Maharashtra (Rural)	2
West Bengal	3
Nepal:	3

Child participants (*N* = 12) ranged in age from 12 to 17 years (mean = 13.9 years). Most (*n* = 7) were male, and all but one were enrolled in school (i.e., online); that child had dropped out 2 years prior in order to receive medical treatment and had not yet returned (see Table 2).

**TABLE 2:** Child demographics.

Variables	Total sample (*N* = 12)
Age: mean (range; SD)	13.9 years (12–17 years; 1.2 years)
Sex:	
Male (*n*)	7
Female (*n*)	5
Children's residence [typical]:	
Hanuman Tekdi	4
Village and Hanuman Tekdi	2
Hostel	6
Children's siblings’ residence:	
Hostel	7
Village	10
School (typically enrolled) (*n*):	10
Years in school:	7.1 years (5–11 years; 2.3 years)
Career aspirations (*n* ^a^):	
Accountant	1
Attorney	1
Cricketer	3
Footballer	1
Government	1
Hotel manager	1
Mechanic	1
Police/law enforcement	3
Singer	1
Do not know	1

^a^ Some identified more than one future career aspiration.

## 6 Results

### 6.1 Mothers' Experiences

#### 6.1.1 Developmental Pathways Leading to Hanuman Tekdi

Although we did not target sex trafficking victims, most participants (*n* = 10) had been trafficked into sex work as minors. Seven of those were forced into the sex trade, and the remaining three were coerced by friends or acquaintances. Indira explained how, at age 15, individuals approached her in her village and lured her away under the pretext of employment in the Bangalore film industry—she would provide water to the actors. “I was never taken to Bangaluru and was brought here in Hanuman Tekdi for this work. I was very frightened in the beginning. I was not ready to do this work. But they forced me to attend customers.” Aisha, also aged 15 when she entered the sex trade, had been living with an aunt who regularly beat her. When a woman wearing “ornaments and good clothes” came to the village, Aisha “…begged her to take me as a housemaid. The lady agreed and landed me in HT. She cheated me. I cried a lot but was helpless.” Similarly, Laila explained “A person known to me promised me of cleaning job in city. He left me in HT for this work. I thought it's written in my destiny to do this.” These stories resonated with all who had been trafficked.

In contrast, nine of their peers did not meet the legal definition of sex trafficking (as laid out in the UN Trafficking in Persons Protocol [UNHRC 2000]). They entered the CSI as adults and out of necessity—they were poverty‐stricken, alone (typically abandoned by a husband), and without kin with financial means to assist (i.e., generations of indigence). They needed a way to support themselves and their children. To illustrate, Veda was 20 years old and had an infant, when her husband left her. She was coerced by promises of a good job by a woman she knew, “But I landed here. It was difficult to accept this as my profession. But I was helpless and accepted.”

#### 6.1.2 Intergenerational Family Dynamics and Child Residence Patterns

##### 6.1.2.1 Intergenerational Family Dynamics

All participants came from deeply impoverished rural areas—where indigence, caste, and gender discrimination set their life in motion, and where subsistence living was passed from one generation to another. In such contexts, gender‐based norms—and expectations of female purity—prevail. Thus, regardless if one was trafficked into the CSI by force or coercion, or entered reluctantly in order to survive, disdain, judgment, and familial exclusion typically resulted. To the extent possible then, participants went to great lengths to conceal their CSI involvement. Twelve participants indicated that their families were not aware of their work in the CSI. Gita's story is illustrative. She was very poor and working at a factory in Nepal. A man offered her employment elsewhere and “guaranteed us better earnings.” Her husband allowed her to go, indicating he would follow shortly with their child. Gita was left in HT and “had no choice but to do the work.” When her husband arrived and learned of her occupation, he demanded a divorce and the son. She refused, telling him “…to go live his life and I will live mine with my son. And thus he left me. One good thing he did was he never told my relatives about my profession.” Thus, she was able to retain good relations with them. Referring to her brothers, Gita noted, “They always welcome me at village. But I never ask for any help to them since they have their own expenses.”

Seven participants indicated that their families were aware of their involvement in the sex trade. Among these, family relations were strained or nonexistent. To illustrate, since learning of her profession, Mona's family refused to acknowledge her. She had not had contact with them in almost two decades, and stated, “I do not know where they are or if they are alive or not.” Likewise, Pushpa explained, “I didn't travel since last 12‐13 years. As [kin] came to know about my profession. They prevent me from visiting them.” Likewise, although Diva's parents were dead she had siblings alive in her native village. Still, she had not returned in over 15 years. Despite being trafficked in the sex trade at age 15, her siblings wanted nothing to do with her. She explained, “A girl staying out of home [alone, without a husband] is not accepted; I don't call them as they do not want to be in touch.” She added, “my brother refused to let my son stay at his place.” Interestingly, two women self‐identified as Devadasi (a caste where sex work is normative and intergenerational), yet they too were alienated from family. Samira is a case in point. Although her parents were deceased, her brother lived in Mumbai, only few miles from HT. Still, she had not seen him since childhood explaining: “He never inquired about me.”

##### 6.1.2.2 Child Residence Patterns

Participants had a total of 37 children (mean = 1.7; range = 1–4 each). As expected from the literature, children resided in a variety of locations, including with their mothers in Hanuman Tekdi (*n* = 10), in a local hostel (*n* = 13), or with kin in the natal village (*n* = 14). Reasons for children's diverse residence patterns varied but seemed intricately connected to two factors: (a) if mothers' extended kin were still alive in natal villages; and (b) if mothers' extended kin were aware of their occupations. Generally speaking, mothers with children living in their natal villages were those whose kin were still alive and whose kin were unaware of work in the sex trade. On the other hand, participants without extended kin in natal villages or those whose kin were alive but knew of their occupations and refused to provide assistance were forced to enroll their children in hostels or keep them in HT.

#### 6.1.3 Present Concerns and Children's Futures

Data were collected in March 2021—in the midst of the global pandemic. Because of the country‐wide lockdown, children's schools and hostels remained closed. Children who normally lived in hostels were instead living with their mothers in HT or staying in villages with kin. Mothers had almost no customers during the COVID‐19 lockdown. Given the context, mothers' concerns largely centered around the need for income, returning children to their hostels, and getting children educated so they were employable. Samira's children were being educated in her natal village and she called them “…every Friday at any cost” and then continued, “Only for my children I will sell myself. [In Hindi: *Sirf mere baccho ke liye khud ko bechungi*]. But I will educate children at any cost.” Chanda echoed this statement, indicating great concern that her children be educated, stating “I will make [children] educated at cost of my life.” Indira similarly noted, “My dream is to educate my child and see him settle down in life. I want to send him abroad for work.” She then remarked, “As soon as [the] lockdown [is] lifted my first priority will be to admit my son into hostel again.” Gita simply said, “My dream is to see my child succeed in his life. [In Hindi: *Mera ek hi Sapna hain, ki mere bacche age badhdke tarakki kare*].”

On the other hand, several participants (*n* = 3) had children who were grown. Mothers also expressed concerns for adult children. For instance, Veda's 20‐year‐old son had quit school. “Now,” she explained, “he works wherever he can find work.” Given the pandemic and reduction in customers, Veda's situation was precarious because, “There is no income for me, and no regular job for my son.”

### 6.2 Children's Experiences

#### 6.2.1 Developmental Pathways Leading to Residence in Hanuman Tekdi

Prior to the lockdown, only four of the 12 children resided in HT. The others lived primarily in hostels (*n* = 6) or shared time between HT and mothers' home villages (*n* = 2). Thus, staying in HT full‐time was new to the majority. When asked if HT was safe, only four—the four who lived there regularly and were used to the environment—responded “yes.” The remaining eight felt differently. Nearly all described the area as “dirty” and all mentioned frequent fighting among neighbors. Anika (15, female) explained: “I feel a little bit dirty. Like every day there is a fight and people swear. They have a bad mouth. I feel a little bit disturbed. [In Hindi: *Gandi bate karte hai, ghin aati hai*].” Parth (13, male) also commented on fighting in the neighborhood—and noted further that his family sometimes also “…fights with the neighbors.” Beyond this, the HT environment in general troubled many. Hardeep (17, male), for instance, stated, “I don't find this place is right. I feel like they are doing all wrong deeds.” His thoughts were echoed by Sanjay (14, male) who explained that he did not mind living in HT, however, he “…does not like the work people do [here].” Dipti (12, female) similarly commented about HT: “No one stays [acts] in a proper way. [In Hindi: *Koi acche se nahi rahta yaha*].”

Without being asked directly, children nonetheless provided insight into strategies their mothers used to keep them safe while in HT. Anika (15, female) reported, for instance, “Mom says the area is not good. She doesn't even allow me to come downstairs. I stay upstairs the whole time.” Likewise, Aanya (15, female), reported that she did not even go shopping because “mom doesn't let me out” instead “I stay at home and do the [house] work.” Ashish (13, female) echoed these comments, saying that she “…stays inside and cooks.” Dipti was supposed to tell her mother if anyone in the neighborhood talked to her. Even under normal conditions, Priti (13, female) spent as little time in HT as possible by finding work (e.g., selling tobacco or working in a bakery). She explained: “Sir, I only go [find work] when I have holidays in school. It's better than me roaming here and there.”

Beyond their own safety, children were also aware that HT posed danger to their mothers. Dev (15, male) stated, “I worry the most about this thing‐ the line of work is not right…[mom] stays in this area. Anything can happen here.” He continued, “I tell mom daily that ‘let's get out of here. You borrow money from anywhere or let's settle in a village. I will do anything for you’.” However, some were also aware that options for other work did not exist. For instance, Aanya (15, female) said, “I feel sad. There is no other source of income so she has to do it [sex work]. She tried getting another job but couldn't find one.”

#### 6.2.2 Informal Social Support: Given and Received

Nearly all participants (*n* = 11) noted having either friends or “best friends.” However, these were typically in their villages, from school, or from their hostels and thus, participants had not seen them since India's lockdown a year earlier. Dev (15, male) explained having “…three best friends in my village. We talk on phone. [But] not so much here [in HT].” Interestingly, some specifically noted the biological sex of their friends. For instance, Samveet (12, male) described having one “best friend” and several other “friends” and specifically indicated “they are all boys.” He also shared, “mom gives them chips.” Likewise, two female children highlighted not having any male friends. Dipti (12, female) was one of these and noted having, “Two best friends, but no male friends. Papa says not to be friends with guys.” Finally, Anika (15, female) mentioned studying with her childhood friend and that they “…will go to the same college.”

Participants were also asked who they talked to when stressed. Not surprisingly, most mentioned mothers as their first source of emotional support. Ashish (13, female) for instance, stated, “I share everything with mummy. She loves me.” Ajay (14, male) commented “first with mom, then with dad”. And Ivaan (14, male) reported being “…closest to my uncle but when I feel stressed I share it with my mom.” Samveet (12, male) talked to friends as well as his mother, but then added, “With mom you can share everything and she can guide you.” Anika (15, female) described her mother as her “best friend” and then said, “I can talk about personal things to my mother.” Similarly, Aanya (15, female) reported sharing “…all things with my mom” and then commented, “She loves me a lot.” However, when she felt “stressed,” Aanya said, “I talk to God.”

When asked what their mothers did for them, or what they asked her for, participants' responses remained consistent. In fact, Hardeep's (17, male) comment that his mother “… provides everything we ask for—like food, clothes, and other things” was echoed in the responses of many. Ajay (14, male) reported that his mother “…cooks food, helps me in studies” and Samveet (12, male) asked his mother “…for chocolate, pencils, erasers, books.” Ashish (15, female) too indicated that her mother “…gives me everything I ask her for. I ask for toys and she gives it. When I ask for money she gives it.” Likewise, Parth (13, male) said his mom, “…gives me anything I ask for—textbook, notebook bag, pencil.”

However, interesting variants also emerged. For instance, when asked if he shared everything with his mother, Sanjay (14, male) replied, “No, I don't share everything with her.” Priti (13, female) reported similarly, “I don't tell everything to mom‐ we tend to share with friends more. If I tell mom she will shout,” and continued, “I feel nice when she speaks well but sometimes she shouts at me. One day she speaks nicely and the next day she might shout.” Dev (15, male) simply said if problems were to arise, he would first go to his father and that he “tells mother very less [in Hindi: *main maa ko jyada nahi batata*].” Finally, Dipti (12, female) stated, “I love my dad the most—and mom a little bit less.” When asked why, she explained, “Because he does a lot of hard work and brings us things to eat. I also love mom. She also does many things to help us.” But then added, “I never ask for money. My mom doesn't have money.”

When asked to describe how they helped their mothers, children's answers largely referred to instrumental support consisting of cooking food, cleaning, and washing and hanging clothes. To illustrate, Anika (15, female), said, “I help my mother the whole day. I wash vessels, clean house, wash clothes, and cook food.” Dipti (12, female) similarly replied, “This morning first I made my bed, swept outside the house, then washed the utensils and then I cleaned the kitchen.” Samveet (12, male) added to the list saying, “…at market I carry things, I also fill the water jug” and then, “if [mom is] sick I massage her head and go with her to the doctor.” Priti (13, female) similarly noted massaging her mother's legs as well as bringing her tea. To the same question, Aanya (15, female) replied, “I do everything she asks me to do.” Aspects of emotional support were mentioned as well. Hardeep (15, male) simply said, “I try to keep her happy,” whereas Parth (13, male) commented, “I give courage to my mom. [In Hindi: *Main maa ko hosla deta hu*].” Finally, financial support was also noted. Two children indicated plans to give the money they earned from participating in the research to their mothers.

#### 6.2.3 Education and Future Aspirations

With the national lockdowns, schools in India were shut down and education shifted to eLearning and on‐line venues. This proved difficult for many. First, none had personal computers so were forced to rely on mobile phones and WhatsApp to access assignments and materials. However, few (*n* = 4) had their own phones. Instead, they shared mobile devices with others (e.g., siblings or parents). These devices were sometimes “hand me downs.” Furthermore, hanging on to phones was sometimes difficult. Dev (15, male) shared that he was able to participate in on‐line learning until his father's phone was stolen from their home. Finally, youth also expressed the difficulty in finding others, at home, who could assist them with homework or studies. Samveet expressed, for instance, that his mother was not educated so he had to rely on his neighbor and best friend for assistance. And all noted the difficulty in “studying” online—without the proper materials (e.g., books, papers).

Despite these challenges, children were deeply aware of the necessity of education. Dipti (12, female) explained, “If we study properly, when we become adults we can get a job in the office. My dad told me to become a police officer.” Similarly, Aanya (15, female) did not like having schools closed. She noted, “We need schooling to grow ahead in life. [In Hindi: *Age badhne ke liye, school jana jaruri hain*].” In terms of the future, several spontaneously indicated plans for college. In fact, Anika (15, female) had just passed the 11th standard exam necessary for college entrance. In terms of specific forms of future employment, children's responses varied considerably—from accountant to singer; although most mentioned police officer (*n* = 3) or professional cricketer (*n* = 3). In the future, Sanjay (a 14‐year‐old male) simply described his desire to “get a good job and take her [mom] far from here.” This sentiment was similarly expressed by Samveet (12, male) who hoped to “get older and get a job—then she [mom] won't have to work here [HT].”

## 7 Discussion

This is the first empirical investigation, of which the authors are aware, to explore in depth the familial and contextual environment of brothel‐based FSWs and their children. Application of ecological systems theory (Bronfenbrenner 1977, 2005) unveiled unique nuances inherent to daily life as they navigate the challenges associated with abject poverty, caste and gender discrimination, stigma, and political systems blind to their plight.

Although this was not an investigation of sex trafficking, per se, the majority of FSW participants were sex trafficking survivors, whereas indigence and lack of alternatives forced their peers into the sex trade. These findings mirror a growing body of literature (Dalla and Kreimer 2017; Joffres et al. 2008; Sinha 2015; Swathisha and Sibnath 2022; Swendeman et al. 2015) which defies typical assumptions of brothel‐based sex workers being “born into brothels” (i.e., to mothers working in the brothel‐based sex trade who simply follow in their mothers' footsteps). Noteworthy here are the myriad macrosystemic forces (gender and caste discrimination, poor infrastructure, and absence of services to assist rural poor) that constrain life options and compel sex work for the majority of FSWs in India (Joffres et al. 2008; Saggurti et al. 2011; Vijayakumar et al. 2019).

Despite circumstances surrounding entry, mothers were adamant that their children would not remain in Hanuman Tekdi or work in the CSI. Two things were necessary to ensure a different route for their children: (a) access to education for upward mobility; and (b) protection from present dangers lurking in the exosystem (i.e., Hanuman Tekdi area). To the first point, mothers in this study—as well as in other studies with comparable samples (Dalla et al. 2019; Sinha and Prasad 2020)—expressed great concern for their children's education. In fact, having children educated and employable in an industry far from the sex trade was FSW participants' top priority. Interestingly, children too expressed a strong desire to be educated—they “missed school” and liked to study. Illustrative of this point too are the things child participants requested from mothers: toys, on the one hand, and textbooks, books, notebook bags, pencils, and erasers, on the other. These are children motivated to learn. Unfortunately, (and again) innumerable macrosystem barriers inhibit educational prospects for India's most marginalized children (Chowdhurry 2022; Gupta and Morris 2022; UNICEF, forthcoming).

To the second point, brothel‐based mothers commonly protect children from ecosystem dangers by enrolling them in hostels—or government‐run boarding schools—to remove them entirely from the red‐light areas (Basu and Dutta 2011; Espana et al. 2021; John‐Fisk 2013; Praimkumara and Goh 2016). Thus, we were expecting (and found) similar results. However, hostel closures for up to 18 months due to COVID necessitated new strategies. Although not asked directly, children nonetheless provided insight into actions taken by mothers to shield them from neighborhood risks. These included, most often, forcing children to stay “inside” and, even more restrictively, forcing them to stay “upstairs” in addition to closely monitoring all interactions with neighbors—adults as well as other children.

The macrosystem (e.g., political and economic systems) is typically described as having significant but indirect influence on child development (Bronfenbrenner 2005). Of note is that during the pandemic, India had the longest school closure (82 weeks) of any country in the world, except Uganda (VOA News 2022). Decisions made in haste at the macrosystemic level, without proper provisions to assist those in greatest need, deeply and directly impacted the microsystems of both FSW mothers and their children. With school closures—two microsystems to which children were accustomed and familiar, namely, hostels and schools—were instantly gone. Many were thrust into an unfamiliar world, where former exosystems (mother's work and the HT environment) were instantly transformed into microsystems—having direct and daily impact on their well‐being and necessitating significant adjustments and resourcefulness. Resilience—among mothers and their children—comes readily to mind.

Direct questions about friends and confidants led to unique insight into children's relationships with informal sources of support. Noteworthy is that researchers often inquire about informal support that is *received*, rarely do they ask about support that is *given*. We did, and children's answers were illuminating. Children described giving and receiving many types of support to mothers, including instrumental, emotional, and financial. Not only were daily routines exposed (e.g., household chores) but so too were deeply felt emotional connections. Unsolicited comments from children, such as “My mom is my best friend” and “I give my mother courage”, paint intricate pictures of the tight bonds between mother and child.

Interestingly too, we learned that, under normal conditions (not during a global pandemic), children's residential patterns appeared quite fluid—with movement from rural village, to hostel, to mother's place in the brothel area—as circumstances demanded. Moreover, children did not necessarily reside with siblings. That is, several children in a sibling string could reside in very different places—one or two in the village, others in a hostel, or with mothers. These are complex family dynamics, and each microsystem houses unique people (e.g., extended family, peers, hostel administrators, parents, and aunts/uncles) and patterns of social interaction that shape child outcomes. However, extended family support is *crucial* for children to have options of residence in their mothers' natal village. This finding parallels the work of Pardeshi and Bhattacharya (2006) who discussed varied types of child‐rearing practices among brothel‐based FSWs dependent upon informal support from extended kin. One critical difference between this study and that of Pardeshi and Bhattacharya involves Devadasi participants. That is, Pardeshi and Bhattacharya (2006) described Devadasi as having strong ties to extended kin in natal villages which provided multiple protective factors, both during and after pregnancy, as well as a safe place—far from the urban red‐light areas—for their children to live and grow. The extant literature mirrors these findings (Gayathri 2014; Gurav et al. 2013; Orchard 2007a, 2007b). However, such was not the case in this study. Our sample included two Devadasi—and neither received assistance from families of origin. Clearly, assumptions cannot be made about the availability of informal kin support.

### 7.1 Implications for Practice, Policy, and Continued Research

This research contributes important information for practice and policy, and for guiding future research with similarly vulnerable populations. First, mothers' strategies for protecting children in dangerous contexts is a unique contribution of this study—and begs the question as to how *formal support* organizations can assist in that task. Although night shelters are commonly available in red‐light brothel areas for keeping children safe overnight, daytime strategies (especially in situations when children are not in school or unable to attend) must be considered as well. Another important area for consideration involves home‐schooling strategies for children in red‐light areas who are unable to attend school. How can formal resources be extended to bring education into children's homes—especially when resources are lacking or nonexistent (e.g., computers, Wi‐Fi or internet access, and educated adults) to assist with and promote home‐based education? Access to devices and broadband is essential as education, health care, and other services pivot to virtual platforms. Ray and Chakravarty (2025) discuss the impact and effectiveness of Mobil School Education Programs in Indian slums. They write:

The initiative is specifically crafted to deliver formal or non‐formal education to marginalized and vulnerable populations, particularly those residing in challenging circumstances such as slums, street communities, or temporary settlements, to improve access to education in these communities. (p.2)

Their evaluation targeted five school bus programs that were strategically selected based on geographic diversity and funding sources (corporate social responsibility initiatives, NGOs) and included semi‐structured interviews with key stakeholders and focus groups with parents. The results are promising. Parents consistently highlighted, “…the transformative impact of mobile schools in making education accessible,” (p. 10). Innovation and adaptability are key to successfully addressing the structural barriers to educational equity within resource‐poor, marginalized communities.

Policy recommendations aimed at similar groups of women and children are extensive—and extensively addressed elsewhere (Dalla et al. 2019; Dasgupta and Sinha 2021; Global Fund for Children‐India, 2019; Goldenberg et al. 2021; Karandikar et al. 2024; Sehgal and Patni 2023; UNHRC 2022). Here, we take a bold stance and say simply: children living in impoverished and resource‐poor areas face tremendous *systemic structural barriers* (e.g., access to quality education, access to safe and affordable housing, food insecurity, lack of clean water and sanitary facilities, crowded living conditions, and community violence), with the additional burden of stigma and discrimination when mothers are associated with the commercial sex industry. Local, state, and federal government agencies must (a) care about and (b) recognize the potential of these children (i.e., talents, skills, and abilities to be developed) if they are to act to reduce or remove those barriers. This is paramount if the vast array of thoughtful, relevant, and practical policy recommendations readily available to those in power are transformed into action.

This research highlighted children's fluid residence patterns in terms of both geography (i.e., urban hostel, rural village, and brothel area) and people (i.e., peers and hostel directors/administrators, extended kin, siblings, mothers, and uncles). Deeper examination of the impacts of these diverse settings, relationships, and living arrangements on well‐being is warranted. Peer relationships with vulnerable youth can be a tremendous source of support and care—as evidenced in this study. Examination of those relationships through time—especially in the absence of extended kin support—would be valuable for better understanding developmental trajectories and sources of resilience among uniquely vulnerable children. Continued investigation of familial dynamics among highly vulnerable and stigmatized populations is warranted—and clearly valuable for mitigating assumptions and eliminating misperceptions.

### 7.2 Limitations

Despite the insights gained, limitations are also inherent in this investigation. First, data were collected in one red‐light brothel area only. Although results may likely reflect what would be found under comparable conditions and similar samples elsewhere in India, caution is advised in making generalizations. Further, our study included a small sample—19 mothers and 12 children. Although saturation was reached, the sample represents but a handful of those estimated to be living in the HT area (e.g., 500 FSWs; GaonConnection 2020). Continued research, with larger samples and including multiple sites, is recommended. Additionally, the sample includes mothers and children only. This was by design given our purposes. However, future work should consider expanding the sample to also include children's teachers, hostel supervisors, and fathers or extended kin, to the extent possible. Finally, the interviews were conducted in Hindi, transcribed, and then translated into English. It is likely that there was some data loss due to this, and some cultural or linguistic meanings may be skewed or missing from the data. On the other hand, engaging in cross‐cultural research with populations who speak a different language relies on translation and would not be otherwise possible. Moreover, sometimes ideas are sparked when engaging with translated material (e.g., it could mean *x* or *y*) that one had not previously considered.

## 8 Conclusion

This research contributes significantly to the literature, specifically on the mother–child relationship by showing examples of how sex workers as mothers are doing the best they can under the circumstances in which they live. Insights from this research have significant implications for social workers and community practitioners working in brothel areas of India in shaping interventions that are children friendly and aim to keep children and their mothers together in a safe and healthy environment. The results of the research highlight the need for government and nongovernmental support on an ongoing basis and, more importantly, during unexpected crises.

Lastly, this research provides eye‐opening evidence on the challenges and difficulties faced by sex workers and their children. However, this is just the beginning—advanced longitudinal research is imperative to dispel myths surrounding the children of sex workers and move away from disparaging labels. Additionally, it is crucial to focus on the agency and decision‐making of sex workers, highlighting their daily efforts to prioritize the well‐being of their children.

## Conflicts of Interest

The authors declare no conflicts of interest.

## Supporting information


Data S1

## Endnotes

^1^
A business establishment where commercial sex workers are available for hire (Merriam‐Webster, n.d.).

^2^
Dalits (formerly ‘untouchables’) constitute approximately 17% of India's total population and include about 1100 unique castes who, collectively, occupy the lowest rung on India's caste hierarchy (Metcalf and Metcalf 2012). Historically, untouchables were denied social, economic, cultural, and political rights, including rights to property and education. Despite the enactment of laws to abolish discriminatory practices against Dalits, caste discrimination remains pervasive across the country (Agrawal 2016).

^3^
This is a caste where participation in the sex industry is intergenerational and normative. Historically, the Devadasis would dedicate a child to a God/Goddess to bring good fortune or protection from illness; these children were revered for their dedication and service to the temple. Sometimes, this included sexual services to temple patrons and priests. Today, the Devadasi tradition is associated with child sexual exploitation (Orchard 2007a, 2007b).

^4^
Compensation amount was based on communication with the research assistant who is actively engaged with sex worker communities in India, and the two lead authors' own extensive work and research with sex workers in India. Based on these assessments, the compensation amount was determined to be appropriate.
