# Supplementary material for: “Anything Can Happen Here”: Mother–Child Experiences Navigating Life as Residents of an Urban Red‐Light Brothel District in India
Source: Fam Process. 2025 Mar 30;64(2):e70034. doi: 10.1111/famp.70034 (PMC11955441; doi:10.1111/famp.70034)
Supplement: Supplementary file 1 — Data S1 [file FAMP-64-0-s001.docx]

**Supplemental Materials**

**Resultant Themes and Sub-Themes**

**Mother’s Experiences**

Theme 1: Reasons for Entry

Theme 2: Intergenerational Family Dynamics and Child Residence Patterns

- Sub-theme 1: Intergenerational Family Dynamics
- Sub-theme 2: Child Residence Patterns

Theme 3: Concerns and Children’s Futures

**Children’s Experiences**

Theme 1: Residence in Hanuman Tekdi

Theme 2: Informal Social Support

- Support Received
- Support Given

Theme 3: Education and the Future
